# Supplementary material for: Risk factors for pulmonary infection in elderly patients with type 2 diabetes: A protocol for systematic review and meta-analysis
Source: PLoS One. 2025 Jul 22;20(7):e0328355. doi: 10.1371/journal.pone.0328355 (PMC12282917; doi:10.1371/journal.pone.0328355)
Supplement: S2 File — (DOCX) [file pone.0328355.s002.docx]

**S2 File Search strategy**

**Taking PubMed, CNKI search strategy as an example.**

| **PubMed** | |
| --- | --- |
| # | Query |
| #1 | "Diabetes Mellitus, Type 2"[Mesh] |
| 2 | (“Diabetes Mellitus, Type 2”[Title/Abstract] OR “Diabetes Mellitus, Adult-Onset”[Title/Abstract] OR “Adult-Onset Diabetes Mellitus”[Title/Abstract] OR “Diabetes Mellitus, Adult Onset”[Title/Abstract] OR “Diabetes Mellitus, Ketosis-Resistant”[Title/Abstract] OR “Diabetes Mellitus, Ketosis Resistant”[Title/Abstract] OR “Ketosis-Resistant Diabetes Mellitus”[Title/Abstract] OR “Diabetes Mellitus, Non Insulin Dependent”[Title/Abstract] OR “Diabetes Mellitus, Non-Insulin-Dependent”[Title/Abstract] OR “Non-Insulin-Dependent Diabetes Mellitus”[Title/Abstract] OR “Diabetes Mellitus, Stable”[Title/Abstract] OR “Stable Diabetes Mellitus”[Title/Abstract] OR “Diabetes Mellitus, Type II”[Title/Abstract] OR “NIDDM”[Title/Abstract] OR “Diabetes Mellitus, Noninsulin Dependent”[Title/Abstract] OR “Diabetes Mellitus, Maturity-Onset”[Title/Abstract] OR “Diabetes Mellitus, Maturity Onset”[Title/Abstract] OR “Maturity-Onset Diabetes Mellitus”[Title/Abstract] OR “Maturity Onset Diabetes Mellitus”[Title/Abstract] OR “MODY”[Title/Abstract] OR “Diabetes Mellitus, Slow-Onset”[Title/Abstract] OR “Diabetes Mellitus, Slow Onset”[Title/Abstract] OR “Slow-Onset Diabetes Mellitus”[Title/Abstract] OR “Type 2 Diabetes Mellitus”[Title/Abstract] OR “Noninsulin-Dependent Diabetes Mellitus”[Title/Abstract] OR “Noninsulin Dependent Diabetes Mellitus”[Title/Abstract] OR “Maturity-Onset Diabetes”[Title/Abstract] OR “Diabetes, Maturity-Onset”[Title/Abstract] OR “Maturity Onset Diabetes”[Title/Abstract] OR “Type 2 Diabetes”[Title/Abstract] OR “Diabetes, Type 2”[Title/Abstract] OR “Diabetes Mellitus, Noninsulin-Dependent”[Title/Abstract]) |
| 3 | #1 OR #2 |
| 4 | “pulmonary infection”[Title/Abstract]) OR “pulmonary infect*”[Title/Abstract]) OR “lung infect*”[Title/Abstract])OR “pneumonia*”[Title/Abstract]) |
| 5 | #3 AND #4 |
| 6 | "Risk Factors"[Mesh] |
| 7 | (“Risk Factors”[Title/Abstract] OR “Factor, Risk”[Title/Abstract] OR “Risk Factor”[Title/Abstract] OR “Population at Risk”[Title/Abstract] OR “Populations at Risk”[Title/Abstract] OR “Risk Scores”[Title/Abstract] OR “Risk Score”[Title/Abstract] OR “Score, Risk”[Title/Abstract] OR “Risk Factor Scores”[Title/Abstract] OR “Risk Factor Score”[Title/Abstract] OR “Score, Risk Factor”[Title/Abstract] OR “Health Correlates”[Title/Abstract] OR “Correlates, Health”[Title/Abstract] OR “Social Risk Factors”[Title/Abstract] OR “Factor, Social Risk”[Title/Abstract] OR “Factors, Social Risk”[Title/Abstract] OR “Risk Factor, Social”[Title/Abstract] OR “Risk Factors, Social”[Title/Abstract] OR “Social Risk Factor”[Title/Abstract]) |
| 8 | #6 OR #7 |
| 9 | #3 AND #5 AND #8 |

| **CNKI** | |
| --- | --- |
| # | Query |
| #1 | 主题 (2型糖尿病+Ⅱ型糖尿病+成人发病型糖尿病+非胰岛素依赖性糖尿病+非胰岛素依赖型糖尿病+二型糖尿病) |
| #2 | 篇关摘 (2型糖尿病+Ⅱ型糖尿病+成人发病型糖尿病+非胰岛素依赖性糖尿病+非胰岛素依赖型糖尿病+二型糖尿病) |
| #3 | 关键词 (2型糖尿病+Ⅱ型糖尿病+成人发病型糖尿病+非胰岛素依赖性糖尿病+非胰岛素依赖型糖尿病+二型糖尿病) |
| #4 | #1 OR #2 OR #3 |
| #5 | 主题 (肺部感染 + 肺炎 + 肺感染) |
| #6 | 篇关摘 (肺部感染 + 肺炎 + 肺感染) |
| #7 | 关键词 (肺部感染 + 肺炎 + 肺感染) |
| #8 | #5 OR #6 OR #7 |
| #9 | 主题 (危险因素+影响因素+预测因素) |
| #10 | 篇关摘(危险因素+影响因素+预测因素) |
| #11 | 关键词 (危险因素+影响因素+预测因素) |
| #12 | #9 OR #10 OR #11 |
| #13 | #4 AND #8AND #12 |
